# Notes on “After-pains” (Extract)

**Published:** 1883-01

**Authors:** 


### NOTES ON "AFTER-PAINS."

ACTEA RAC.—Crampy pains in groins; pains cause flushing of face; predisposition to neuralgia, which seems to be reflex from uterine disorders.

CAULOPH.—Pains spasmodic, felt in uterus, bladder, and reflex pains in chest and back; patient nervous, weak, sleepless.

CUPRUM.—In tedious after-pains in those who have had many children.

USTIL AGO.—Lochia too profuse, partly fluid and partly clotted; prolonged bearing-down pains; uterus feels as if drawn into a knot.

XANTHOX.—Pains excruciatingly severe; pains extend down along genito-crural nerves.—Dr. Farrington in *Am. Jour. of Obstetrics, etc.*

